# Hydrolysis and condensation of monobutyltin chloride: reaction process analysis with DFT†

**DOI:** 10.1039/d3ra06256b

**Published:** 2023-10-19

**Authors:** Jingwei Zhu, Jianliang Mo, Guohua Shi, Qiying Liu, Gang Xu, Gaorong Han, Yong Liu

**Affiliations:** a School of Materials Science and Engineering, Zhejiang University Hangzhou 310058 China liuyong.mse@zju.edu.cn +86 571 87951842 +86 571 87951842; b Weihai CNG New Materials Technolgy R&D Co. Ltd. Weihai China 264299; c Shanxi-Zheda Institute of Advanced Materials and Chemical Engineering Taiyuan 030001 China

## Abstract

As the initial process of preparing transparent conductive oxide materials from monobutyltin chloride (MBTC) to tin oxide, the hydrolysis and condensation of MBTC to form a dimer Sn_2_ play a critical role. However, the specific mechanism of this process is still unclear. Here we develop a step-by-step searching method based on density functional theory calculation and empirical chemical criteria to determine possible reaction pathways and reveal the most likely reaction mechanism. The wave function analyses of various intermediate species provide more insights into the changes of atomic charge population, chemical bond strength, and coordination situation of central tin in the reaction process. Further investigation on the ring-containing Sn_2_ reveals the existence of unique three-center four-electron (3c–4e) interactions to stabilize the four-membered Sn_2_O_2_ ring structure, which serves as the true driving force for dimerization reaction. These results provide a more detailed understanding of the hydrolysis and condensation process of MBTC and would be helpful for the future optimization of the preparation process of tin oxide films.

## Abstract 2

A step-by-step searching method is adopted to find the reaction pathway connecting MBTC and Sn_2_. Meanwhile, three-center four-electron (3c–4e) interactions within Sn2 are revealed.

## Graphical abstract

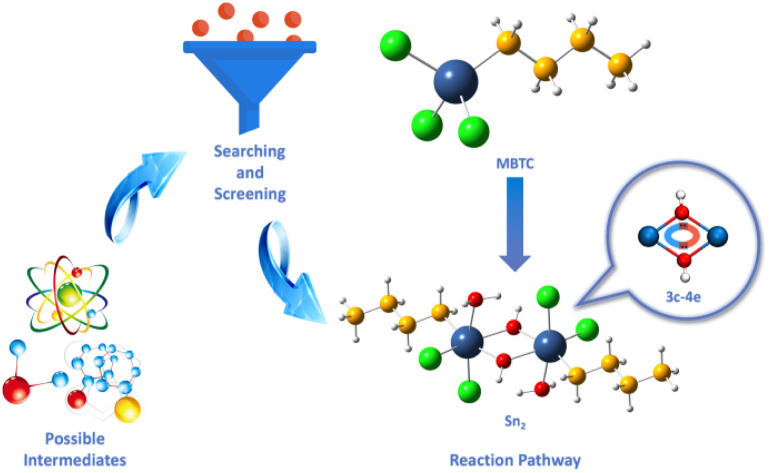

## 1. Introduction

Since first being investigated in 1907, transparent conductive oxide (TCO) materials, which allow light transmittance and electrical current conduction simultaneously, have played an increasingly important role in information display and energy fields.^1^ Among so many TCO materials, tin oxide (SnO_2_) film is widely used due to its advantages of high chemical stability, comprehensive sources of raw materials, and low preparation cost.^2,3^ The quality of tin oxide films and their economics are dominated by process conditions and the precursor system used. Due to its easy storage and low toxicity, monobutyltin chloride (MBTC) is one of the most popular precursors for preparing tin oxide films.

The synthesis of tin oxide from MBTC can be successfully realized using chemical vapor deposition (CVD) and sol–gel routes.^4^ Drawing upon prior extensive investigations, conceivable mechanisms governing the CVD of tin oxide from MBTC have been proposed.^5–9^ While in sol–gel chemistry, the chemical transformation of MBTC follows a distinct reaction pathway. Before the final conversion to tin oxide, MBTC will undergo several stages of hydrolysis and condensation. A great endeavor has been made to understand the hydrolysis and condensation mechanism of MBTC and identify the intermediates in this process. The complete hydrolysis of MBTC is believed to lead to the formation of the stannic acid [BuSn(O)OH]_*n*_, which is considered to be polymeric.^10^ The similarity of resonance positions of [BuSn(O)OH]_*n*_ with those observed for the butyl-tin dodecamer [(BuSn)_12_O_14_(OH)_6_]^2+^ (known as Sn_12_) suggests that stannonic acid belongs to a similar structural type as that of the former.^11–13^ However, the structure of this compound is as yet unclear. While for the partial hydrolysis of MBTC, the product (BuSn)_2_(OH)_2_Cl_4_(H_2_O)_2_ (known as Sn_2_) has been isolated and characterized by X-ray diffraction.^14,15^ Kenane *et al.*^16^ explored this process from a thermodynamic perspective based on density functional theory (DFT) calculations. Their results indicated a seemingly contradictory phenomenon: Sn_2_ formation is favorable at room temperature, but its prerequisite, the hydrolysis of Cl ligand, is highly unfavorable, implying that some elementary reactions and intermediates remain uncovered.

Chemical reactions occur through a series of elementary steps, and a comprehensive understanding of the elementary reactions and intermediate species involved in the hydrolysis and condensation process of MBTC is the key to forming the reaction pathway and revealing the reaction mechanism, which will significantly help optimize the preparation process of tin oxide films. However, studying the hydrolysis and condensation process's mechanism is very challenging since the conversion from MBTC to Sn_2_ is so fast to reach the end that the residual time of intermediates is extremely short. Meanwhile, the current technical conditions limit the direct identification and characterization of structures and compositions of compounds at the molecular level.

In the present work, with the help of DFT theoretical calculations, we developed a step-by-step searching method, starting from MBTC and screening possible intermediates and elementary reactions based on thermodynamic feasibility and some criteria generated from chemical experience and knowledge progressively. Further wave function analyses upon the revealed reaction process, including Mulliken atomic charge and Mayer bond order, have been carried out and discussed in detail, bringing insight into the chemical essence of the process and finding the unique three-center four-electron (3c–4e) interactions in Sn_2_ which might provide the critical driving force in the reaction process.

## 2. Computational details and methodologies

All density functional theory (DFT) calculations were carried out by Gaussian 16 program^17^ here. The popular B3LYP exchange–correlation functional^18^ in conjunction with 6-31+G(d,p)^19–25^ basis set for small atoms (H, C, O, and Cl), and pseudopotential basis set, Stuttgart+ECP,^26^ for Sn were used for geometry optimizations and frequency analyses. After full geometry relaxation and confirming that no imaginary frequencies were found, single point energy calculations were performed using B3LYP with def2-TZVP basis set^27^ for small atoms and Stuttgart+ECP for Sn. Grimme's dispersion corrections with Becke-Johnson damping (D3BJ) were used to describe weak interactions rationally.^28^ In order to simulate the real solution environment, we adopted PCM implicit solvent model^29–31^ with methanol as solvent consistent with the actual experiments.^32^ The Gibbs free energy at 298.15 K and 1 M was calculated as the sum of the single point energy and the thermal correction to free energy obtained from the frequency analysis, while the Gibbs free energy of the proton in aqueous solutions was obtained following the method in the literature.^33^ The reaction Gibbs free energy change Δ*G* was defined as the difference between the total free energy of reaction products and reactants.

The atomic charge is one of the most straightforward and intuitive descriptions of charge distribution in chemical systems,^34^ helping researchers study the states of atoms in various chemical environments and predict reaction sites. The Mulliken atomic charge^35–38^ was employed here for the population analysis. The Mayer bond order^39^ was used to discuss chemical bond strength, which is suitable for judging whether a chemical bond is formed. The Mayer bond order can be understood physically as the number of electron pairs shared between atoms. The higher the Mayer bond order value, the stronger the chemical bond strength for the same chemical bond type. All wave function analyses were carried out with Multiwfn,^40^ which is open-source and freely available. The def2-TZVP basis set was employed for all atoms when generating electronic wave functions.

## 3. Result and discussion

### 3.1. Calculation method validation

**Fig. 1:**
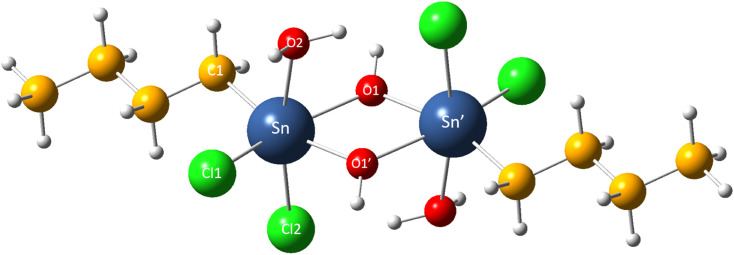
The molecular structure of Sn_2_.

We first validate the selected DFT calculation level to ensure it reasonably reflects the molecular structure and free energy of organotin compounds in the study. The theoretically predicted gas phase geometry of Sn_2_ is shown in Fig. 1 and Table 1. One can see that even though the theoretical bond lengths are overestimated compared with the experimental values, the relative deviations are less than 3%, which is generally within the typical range of error at this level of theory.^41^ On the other hand, the calculated bond angle values are in excellent agreement with the experimental data available,^14^ indicating the reliability of the calculation level employed in the present work.

**Table 1:** Selected bond lengths (Å) and angles (°) in Sn_2_

		Experimental^14^	Theoretical	Relative deviation (%)
Bond length	Sn–O1	2.05	2.11	2.93
	Sn–O1′	2.17	2.20	1.38
	Sn–Cl1	2.42	2.45	1.23
	Sn–Cl2	2.48	2.49	0.40
	Sn–C1	2.12	2.16	1.89
Bond angle	O1′–Sn–C1	162.90	162.90	0.00
	O1–Sn–O1′	69.60	68.70	1.29
	O1–Sn–O2	81.70	81.60	0.12

Moreover, we take a series of R–Sn bond dissociation energies (BDEs) as approximate benchmarks to assess the validity of the selected methodology to evaluate energies. The BDEs, governed by eqn (1), were calculated at 298.15 K as 65.44 kcal mol^−1^, 60.48 kcal mol^−1^, and 56.88 kcal mol^−1^ for R = methyl, ethyl, and isopropyl, respectively, consistent with experimental values (64.00 kcal mol^−1^, 60.00 kcal mol^−1^, and 55.40 kcal mol^−1^),^42^ which again demonstrates that the calculation level is reasonable.

1 (CH_3_)_3_Sn–R → (CH_3_)_3_Sn˙ + R˙

### 3.2. Reaction process of Sn_2_ formation

Hydrolysis and condensation of MBTC in an aqueous solution to form Sn_2_ are supposed to include multiple elementary reactions and intermediate species, potentially hundreds of chemical species and elementary reactions of interest, at least including the following types:

(i) Hydroxyl ion or water molecule nucleophilic attacks central tin atom to form a complex;

(ii) Proton electrophilic attacks high electronegativity ligand;

(iii) Heterolysis of the chemical bond between central tin and ligand, including Sn–O bond and Sn–Cl bond;

(iv) Dimerization reaction.

Since the Sn–C bond is relatively stable in the solution phase, its cleavage is not considered. For computation resources and time-consuming, three criteria based on chemical knowledge and experience were employed here to screen out unreasonable reactions and intermediates:

(1) The acceptable elementary reaction should be feasible thermodynamically, meaning its Δ*G* should be negative;

(2) The reasonable coordination number of Sn in organotin compounds fall in the range of 4 to 6 based on the previous reports;^43,44^

(3) Only water molecule, proton, or hydroxyl ions will be considered to participate in the hydrolysis and condensation of MBTC, ignoring other solvent molecules and species in the solution system as the reactants of elementary reaction.

Another thing to note is that only those hydrolyzed reactants (at least one chlorine atom ligand is substituted by hydroxyl ligand) whose coordination number of central tin does not exceed five were considered capable of dimerization in order to avoid the violation of the above criteria since the coordination number of the central tin atom will increase after the dimerization reaction.

**Fig. 2:**
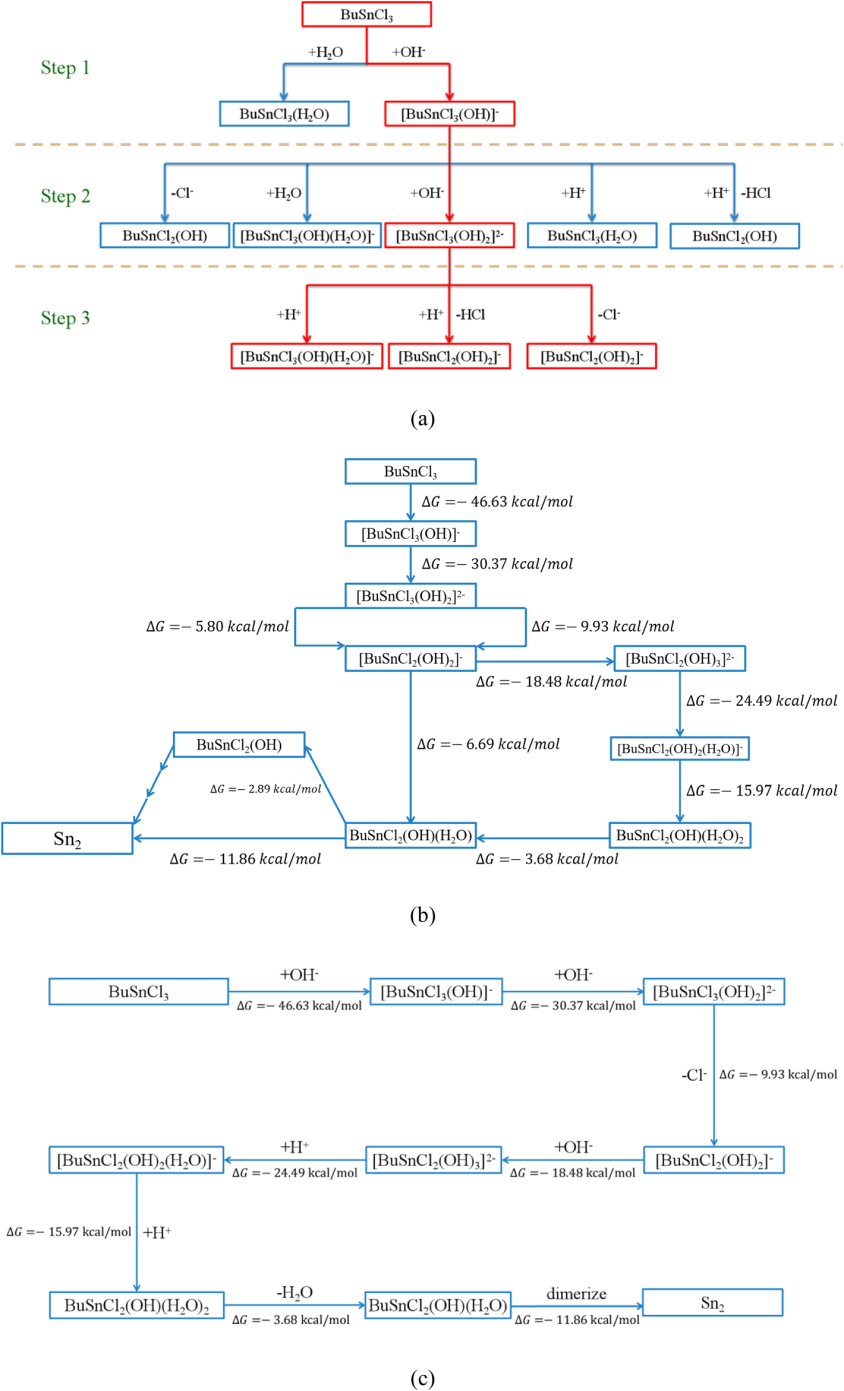
(a) The possible reaction pathway in the first three steps, the process will proceed along the thermodynamically feasible direction (marked in red) (b) all reaction pathways connecting MBTC and Sn_2_ (c) the most thermodynamically favorable reaction pathway of hydrolysis and condensation of MBTC.

Even following the above criteria, more than one hundred possible elementary reactions involving twenty-four species would be considered, which are summarized in Tables 2 and S1–S18.† Taking the first three steps, for instance, as shown in Fig. 2(a), every possible elementary reaction and corresponding Δ*G* were explored, as shown in Table 2.

**Table 2:** Possible elementary reactions and corresponding Δ*G* (kcal mol^−1^) in the first three steps. And the thermodynamically feasible reactions are marked with an asterisk

Possible reaction	Δ*G*
**Step 1**	
*BuSnCl_3_ + OH^−^ → [BuSnCl_3_(OH)]^−^	−46.63
BuSnCl_3_ + H_2_O → BuSnCl_3_(H_2_O)	4.09

**Step 2**	
[BuSnCl_3_(OH)]^−^ → BuSnCl_2_(OH) + Cl^−^	10.17
*[BuSnCl_3_(OH)]^−^ + OH^−^ → [BuSnCl_3_(OH)_2_]^2−^	−30.37
[BuSnCl_3_(OH)]^−^ + H_2_O → [BuSnCl_3_(OH)(H_2_O)]^−^	6.17
[BuSnCl_3_(OH)]^−^ + H^+^ → BuSnCl_3_(H_2_O)	0.95
[BuSnCl_3_(OH)]^−^ + H^+^ → BuSnCl_2_(OH) + HCl	6.04

**Step 3**	
*[BuSnCl_3_(OH)_2_]^2−^ + H^+^ → [BuSnCl_3_(OH)(H_2_O)]^−^	−19.38
*[BuSnCl_3_(OH)_2_]^2−^ + H^+^ → [BuSnCl_2_(OH)_2_]^−^ + HCl	−5.80
*[BuSnCl_3_(OH)_2_]^2−^ → [BuSnCl_2_(OH)_2_]^−^ + Cl^−^	−9.93

Based on the calculated Δ*G* from Table 2, the reactions of hydroxyl ions continuously attacking MBTC could be established since only one possible reaction in Step (1) and Step (2) shows negative Δ*G*:

2 BuSnCl_3_ + OH^−^ → [BuSnCl_3_(OH)]^−^

3 [BuSnCl_3_(OH)]^−^ + OH^−^ → [BuSnCl_3_(OH)_2_]^2−^

However, for Step (3), [BuSnCl_3_(OH)_2_]^2−^ has more than one possible reaction pathway, including electrophilic attack and bond cleavage. [BuSnCl_2_(OH)_2_]^−^ and [BuSnCl_3_(OH)(H_2_O)]^−^, the two products of these possible reactions, were then taken as the starting point for the next step, respectively. Following the above procedure, all possible reaction pathways could be established step by step (see Fig. S2†). Interested in the formation mechanism of Sn_2_, we extracted all reaction pathways connecting MBTC and Sn_2_, as shown in Fig. 2(b). Since the reactions tend to proceed along the path with a more negative value of Δ*G* from the thermodynamic perspective, the most likely reaction pathway could be summarized as the following and shown in Fig. 2(c):

4 [BuSnCl_3_(OH)_2_]^2−^ → [BuSnCl_2_(OH)_2_]^−^ + Cl^−^

5 [BuSnCl_2_(OH)_2_]^−^ + OH^−^ → [BuSnCl_2_(OH)_3_]^2−^

6 [BuSnCl_2_(OH)_3_]^2−^ + H^+^ → [BuSnCl_2_(OH)_2_(H_2_O)]^−^

7 [BuSnCl_2_(OH)_2_(H_2_O)]^−^ + H^+^ → BuSnCl_2_(OH)(H_2_O)_2_

8 BuSnCl_2_(OH)(H_2_O)_2_ → BuSnCl_2_(OH)(H_2_O) + H_2_O

9 2BuSnCl_2_(OH)(H_2_O) → Sn_2_

### 3.3. Wave function analyses of the reaction process

**Fig. 3:**
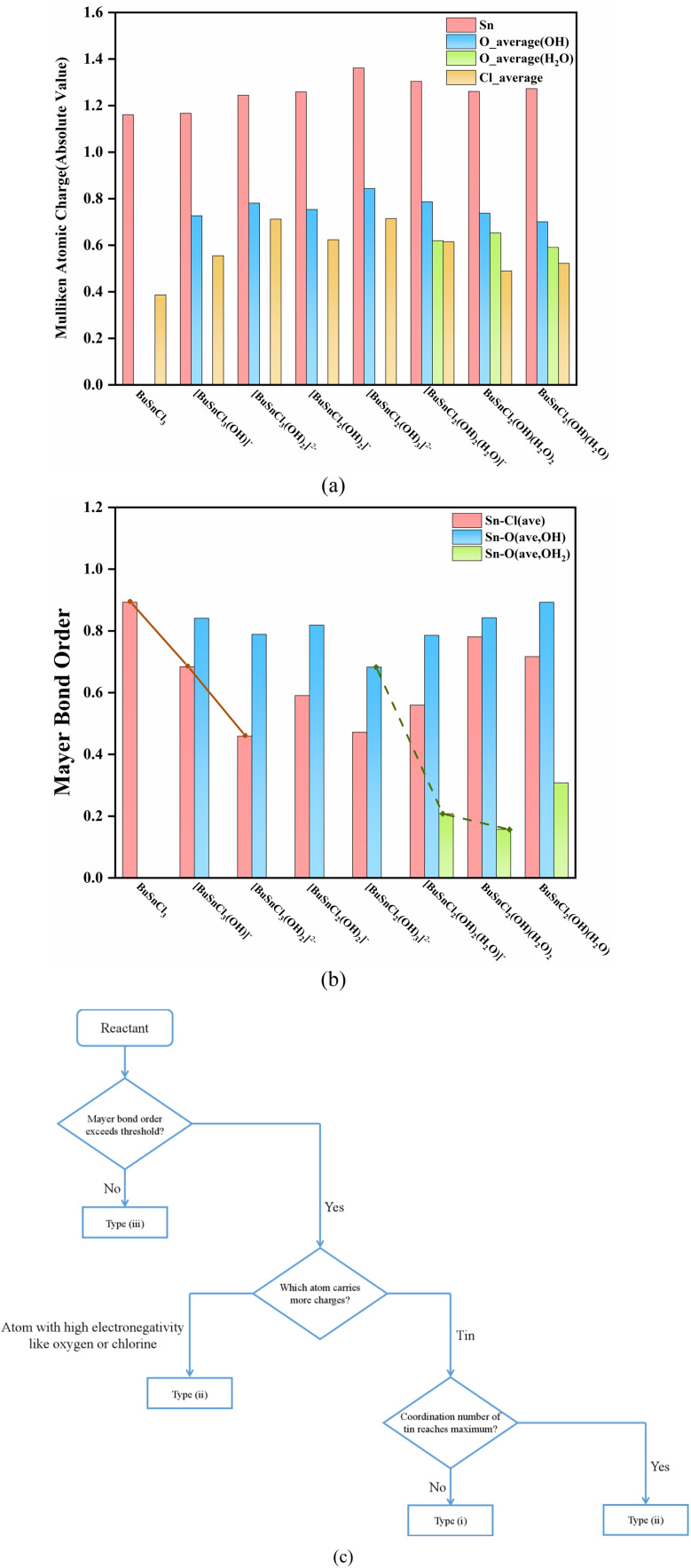
(a) The absolute value of Mulliken atomic charge of species participating in the reaction process. Oxygen is distinguished into hydroxyl oxygen and water oxygen based on different chemical environments (b) Mayer bond order of species participating in the reaction process. Sn–O bond is distinguished into Sn–OH bond and Sn–OH_2_ bond based on different chemical environments of oxygen (c) empirical method of preliminary determining the reaction direction.

The Mulliken atomic charge population and Mayer bond order analysis results of species participating in the reaction process are summarized in Fig. 3(a), (b), and Tables S19–S26,† while the molecular structures and atomic labels of these species are shown in Fig. S3.†

A trend can be observed throughout the reaction process: the process always proceeds with hydroxyl ion nucleophilic attacking the tin atom (Steps (1), (2), and (4)) when the coordination number of the central tin atom has not reached six. That may be due to the fact that the tin carries more atomic charges than oxygen and chlorine, as seen in Fig. 3(a).

When the coordination number of the central tin atom reaches maximum, the reactions in which these compounds serve as reactants all fall in the type (ii) or (iii), as mentioned in Section 3.2. For reaction type (ii), proton electrophilic attacking hydroxyl oxygen atom is preferred (Steps (5) and (6)). Similarly, this can be attributed to oxygen carrying more negative charges than chlorine, as illustrated in Fig. 3(a). As for reactions of type (iii) (Steps (3) and (7)), the breaking of the chemical bond indicates that the bond strength is too weak to be regarded as bonding, reflected at low Mayer bond order values (the value of weakest Sn–Cl bond in [BuSnCl_3_(OH)_2_]^2−^ is 0.32 while weakest Sn–O bond in BuSnCl_2_(OH)(H_2_O)_2_ is only 0.11 as displayed in Tables S21 and S25†). Since the Mayer bond order of the weakest Sn–Cl bond in [BuSnCl_3_(OH)]^−^ and the weakest Sn–O bond in [BuSnCl_2_(OH)_2_(H_2_O)]^−^ are 0.58 and 0.21, we may select Mayer bond order of 0.4 and 0.2 as thresholds for Sn–Cl bond and Sn–O bond to judge whether bonding. Considering that the bonding ability of an atom is limited, the formation of new bonds in nucleophilic or electrophilic attacking reaction will lead to a decrease in the strength of other bonds, as shown by the solid and dashed lines in Fig. 3(b), which is the reason for the generation of low Mayer bond order values.

During the entire process before dimerization, reactions of types (i), (ii), and (iii) occur alternately, which seems irregular. However, as discussed above, multiple factors affect the reaction pathway jointly. The occurrence of reactions of type (iii) is related to the weak strength of chemical bonds, manifested by the low Mayer bond order values. As for types (i) and (ii), the electrical properties of atoms carrying more atomic charges determine which type of reactions will take priority since these atoms are more likely to become reaction sites, while the coordination number of the tin atom should be taken into account because it cannot increase without limit owing to steric effect. Finally, we developed an empirical method to determine the reaction direction, as shown in Fig. 3(c).

**Fig. 4:**
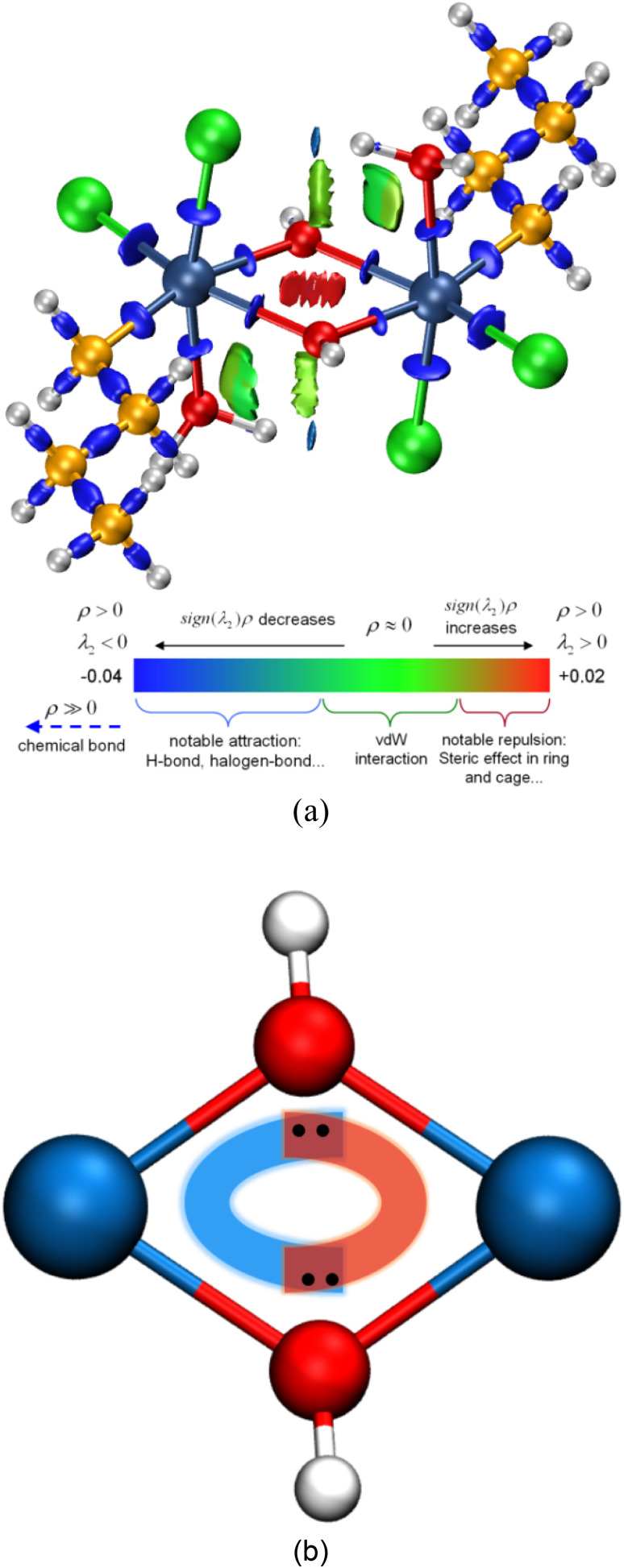
(a) Isosurface map of IRI = 0.8 of Sn_2_. The standard coloring method and chemical explanation of sign(*λ*_2_)*ρ* on IRI isosurfaces are also shown at the bottom, where sign(*λ*_2_) is the sign of the second largest eigenvalue of the Hessian matrix of *ρ*. (b) The interactions within the Sn_2_O_2_ unit of Sn_2_.

For a better insight into Sn_2_, the interaction region indicator (IRI) function,^45^ which is an improvement on the currently popular reduced density gradient (RDG) method,^46^ is employed to clearly show the type, intensity, and position of interactions in Sn_2_. The IRI is defined as follows:10
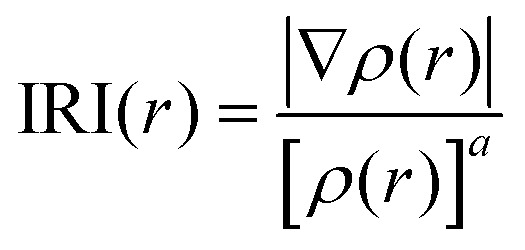
where *a* is an adjustable parameter, and the value of 1.1 is adopted here for the standard definition of IRI. *ρ*(*r*) is the electron density, and ∇*ρ*(*r*) is the electron density gradient. The most suitable isosurface values for IRI in most systems to exhibit various interactions are 0.8–1.1,^45^ and Fig. 4(a) shows the 0.8 IRI isosurface of Sn_2_ coloring based on sign(*λ*_2_)*ρ*, where sign(*λ*_2_) is the sign of the second largest eigenvalue of the Hessian matrix of *ρ* and can effectively distinguish attractive and repulsive interactions.^46^ The region with high electron density and thus large sign(*λ*_2_)*ρ* indicates a relatively strong interaction, while the region showing low electron density and thus small sign(*λ*_2_)*ρ* does not participate in an interaction, or the interaction can be at most attributed to the weak vdW interaction. The blue areas in Fig. 4(a) clearly illustrate the Sn–C, Sn–Cl, and Sn–OH_2_ chemical bonds. Meanwhile, four Sn–OH bonds are also shown in the four-membered Sn_2_O_2_ ring unit. The green areas indicate weak interactions between bridging hydroxyl oxygen and butyl carbon. While the red region located in the center of the Sn_2_O_2_ unit suggests that the electrons are enriched here and exhibit a specific repulsive interaction.

However, the information above also means that each oxygen in the Sn_2_O_2_ unit is tricluster, which deviates from the common understanding. To detect previously unnoticed interactions present in the Sn_2_O_2_ unit, we not only investigate the Mayer bond order of Sn–O1 and Sn–O1′ bonds but also calculate the three-center bond order of O1–Sn–O1′ and Sn–O1–Sn’ (only half of Sn_2_ is considered due to the symmetry), as shown in Table 3. In some sense, the multi-center bond order (MCBO), also known as the multi-center index (MCI)^47^ may be viewed as an extension of the Mayer bond order to multi-center cases. Three-center bond order is defined as:11
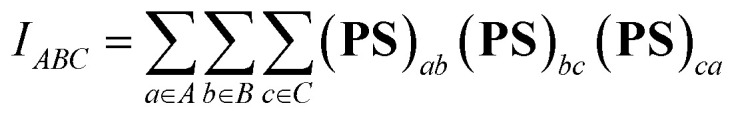


**Table 3:** Mayer or Three-center bond order of selected interactions in Sn_2_O_2_ unit

	Bond order
Sn–O1	0.499
Sn–O1′	0.341
O1–Sn–O1′	−0.243
Sn–O1–Sn′	0.251

As seen in Table 3, the Mayer bond orders of Sn–O1 and Sn–O1′ bonds are still above the threshold, proving the formation of Sn–O bonds in the Sn_2_O_2_ unit. For three-center cases, the bond orders of O1–Sn–O1′ and Sn–O1–Sn′ show negative and positive values, respectively, corresponding to three-center four-electron (3c–4e) and three-center two-electron (3c–2e) interactions.^48,49^ Based on these results, we can infer the reaction process of dimerization: The hydroxyl oxygen atom in BuSnCl_2_(OH)(H_2_O) is bonded to Sn and H atoms through sp^3^ hybridization, leaving two pairs of lone pair electrons. When two BuSnCl_2_(OH)(H_2_O) molecules are in the appropriate position, the hydroxyl oxygen atom in one molecule shares one lone-pair electron with the central tin atom in another to form a coordination covalent bond. Moreover, the remaining lone pair electrons will form 3c–4e interactions in the Sn_2_O_2_ unit to stabilize this structure, as shown in Fig. 4(b), serving as the true driving force for the dimerization reaction whose reaction products possess ring-containing structure.

## 4. Conclusion

Herein, based on DFT calculation results and criteria originating from the chemical experience and knowledge, we developed a step-by-step searching method and identified the most likely reaction mechanism of hydrolysis and condensation of MBTC. It is worth noting that in our calculations, solvent methanol molecules were not considered as reactants. Incorporating solvent molecules into the reaction system undoubtedly leads to more complex reaction mechanisms, which may serve as our future research direction. Through wave function analyses of various species involved in the proposed reaction process, we found that many factors, including atomic charge population, chemical bond strength (Mayer bond order of 0.4 and 0.2 as thresholds for Sn–Cl bond and Sn–O bond), and coordination situation of central tin, control the reaction process. Reasonably considering these factors, we inferred a simple empirical method to preliminary predict the reaction direction. The further exploration of chemical interactions in Sn_2_ provided us with a more comprehensive understanding of the dimerization reaction. During the dimerization reaction, three-center four-electron (3c–4e) interactions are generated within the four-membered ring in Sn_2_ to stabilize this structure which may be the true driving force for this reaction. The results gave a deeper insight into the hydrolysis and condensation process of MBTC, which may be helpful for the future optimization of the preparation process of tin oxide films and provide inspiration for understanding the reaction mechanism of MBTC in the CVD process.

## Conflicts of interest

There are no conflicts to declare.

## Supplementary Material

RA-013-D3RA06256B-s001
