# Supplementary material for: Hydrolysis and condensation of monobutyltin chloride: reaction process analysis with DFT
Source: RSC Adv. 2023 Oct 19;13(44):30718–25. doi: 10.1039/d3ra06256b (PMC10585613; doi:10.1039/d3ra06256b)
Supplement: RA-013-D3RA06256B-s001 [file RA-013-D3RA06256B-s001.pdf]

Electronic Supplementary Material (ESI) for RSC Advances.
This journal is © The Royal Society of Chemistry 2023

Supplementary Information

The Mulliken atomic charge of an arbitrary atom *A* is defined as:

$$q_A = Z_A - \sum_i n_{i,A} = Z_A - \sum_i \eta_i \sum_{a \in A} \Theta_{i,a} \tag{S1}$$

where Z_A is the number of nuclear charges of atom *A*, $n_{i,A}$ is the contribution of atom *A* to occupation number of orbital *i*, $\Theta_{i,a}$ is the population of basis function *a* in orbital *i*, and η is the orbital occupation number.

The Mayer bond order between atoms *A* and *B* is defined as:

$$I_{AB} = \sum_{a \in A} \sum_{b \in B} \left[(PS)_{ba} (PS)_{ab} + (P^S S)_{ba} (P^S S)_{ab} \right] \tag{S2}$$

where **P**, $\mathbf{P}^S$, and **S** are the total density matrix, spin density matrix, and overlap matrix, respectively, while *a* and *b* are basis functions.

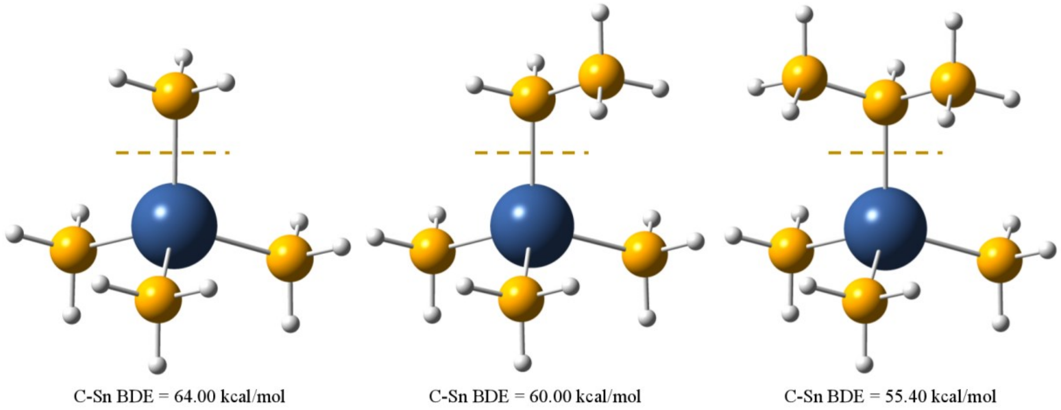

Figure S1. Schematical diagrams of the structures of organotin compounds with the known experimental BDEs of the C-Sn bond.

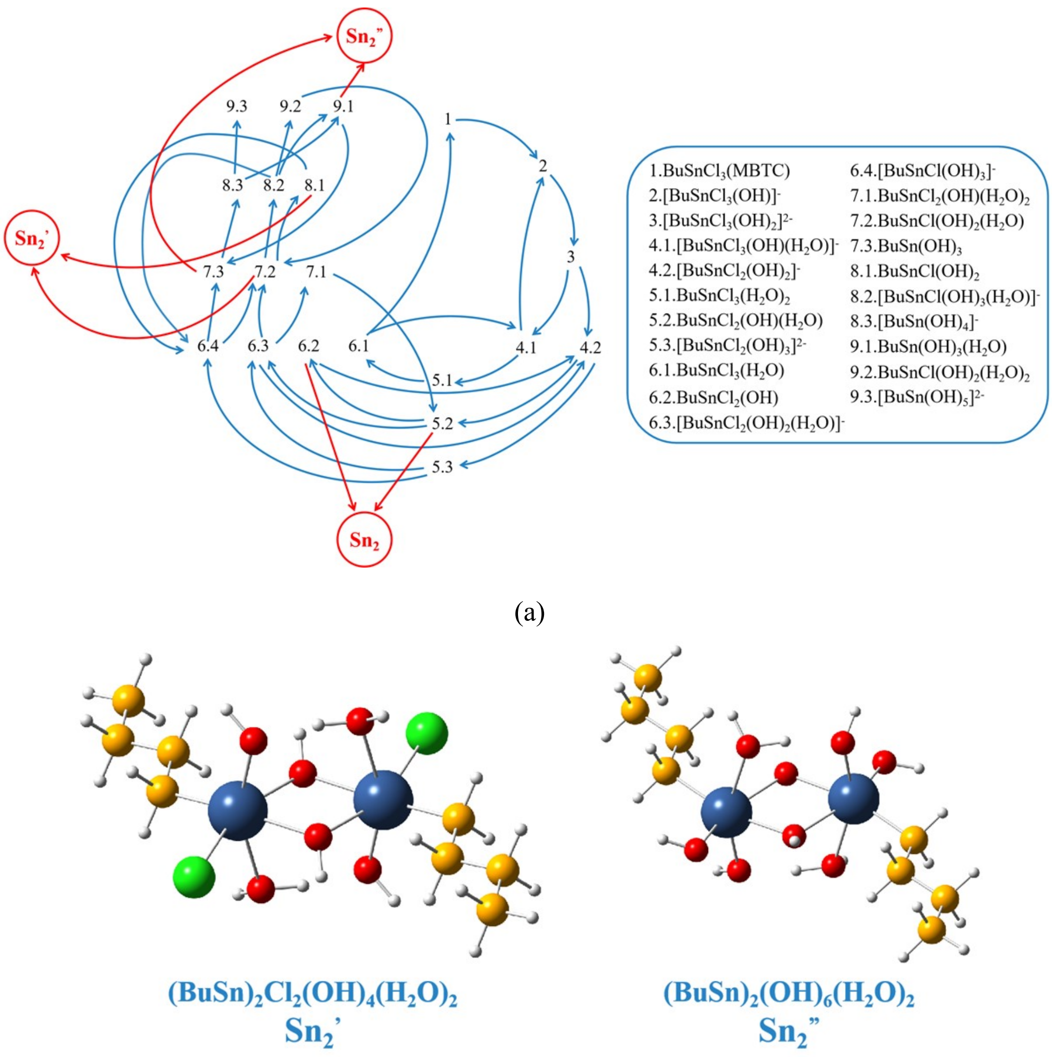

(a)

(b)

Figure S2. (a) All species and feasible reactions considered in the present work. The species are labeled as numbers for convenience, and the corresponding relationship is also shown in the figure. Sn_2' and Sn_2'' are hydrolysis products of Sn_2. (b) The molecular structures and formulas of Sn_2' and Sn_2''.

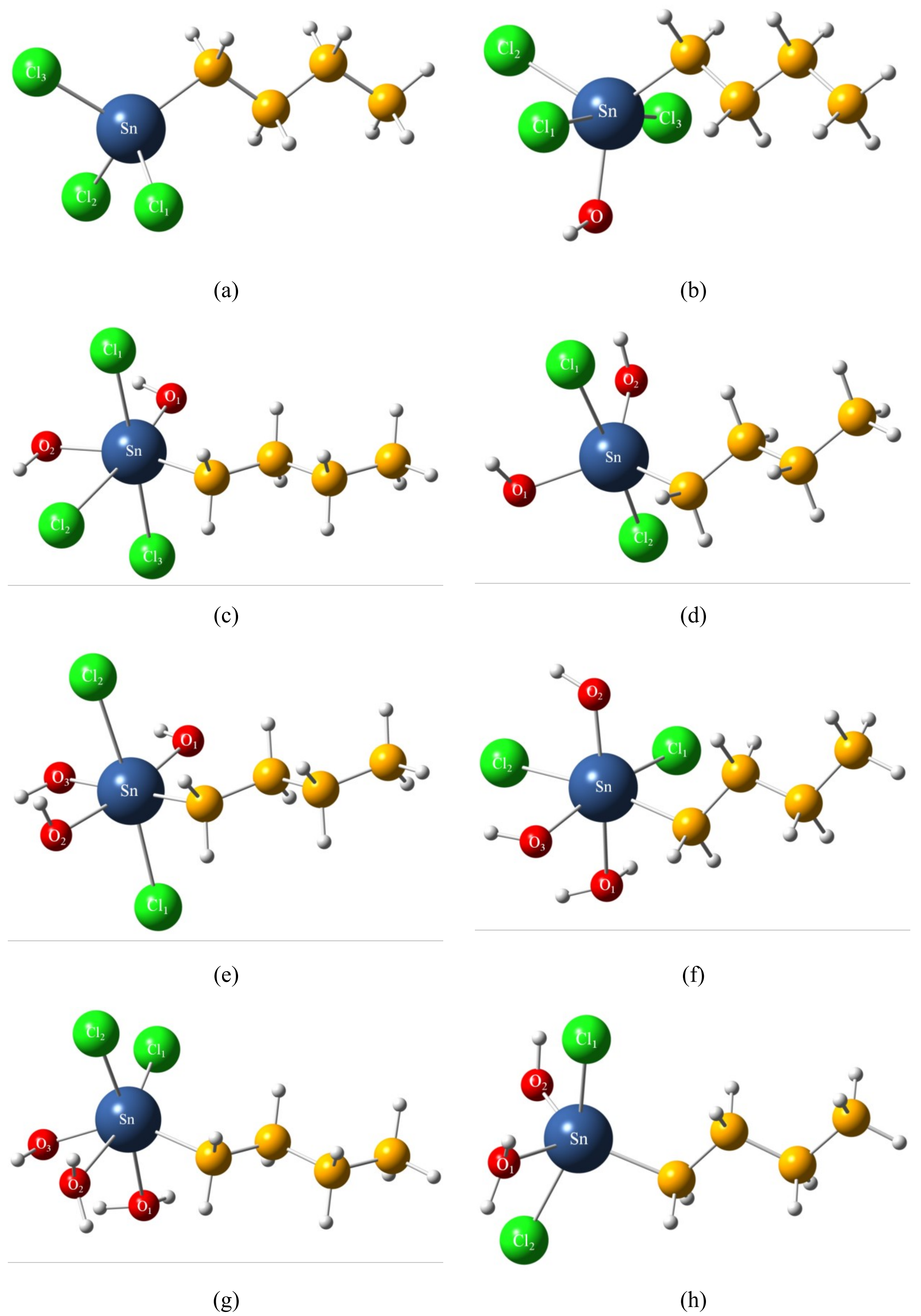

Figure S3. The molecular structures and atomic labels of (a) $BuSnCl_3$ (b) $[BuSnCl_3(OH)]^-$ (c) $[BuSnCl_3(OH)_2]^{2-}$ (d) $[BuSnCl_2(OH)_2]^-$ (e) $[BuSnCl_2(OH)_3]^{2-}$ (f) $[BuSnCl_2(OH)_2(H_2O)]^-$ (g) $BuSnCl_2(OH)(H_2O)_2$ (h) $BuSnCl_2(OH)(H_2O)$.

Table S1. Possible elementary reactions and corresponding Δ*G* (kcal/mol) in Step 4.1.

Step 4.1	
Reaction	Δ*G*
$[BuSnCl_3(OH)(H_2O)]^- \rightarrow [BuSnCl_3(OH)]^- + H_2O$	-6.17
$[BuSnCl_3(OH)(H_2O)]^- \rightarrow BuSnCl_3(H_2O) + OH^-$	44.55
$[BuSnCl_3(OH)(H_2O)]^- \rightarrow BuSnCl_2(OH)(H_2O) + Cl^-$	2.76
$[BuSnCl_3(OH)(H_2O)]^- + H^+ \rightarrow BuSnCl_3(H_2O)_2$	-2.15
$[BuSnCl_3(OH)(H_2O)]^- + H^+ \rightarrow BuSnCl_2(OH)(H_2O) + HCl$	6.89

Table S2. Possible elementary reactions and corresponding Δ*G* (kcal/mol) in Step 4.2.

Step 4.2	
Reaction	Δ*G*
$[BuSnCl_2(OH)_2]^- \rightarrow BuSnCl_2(OH) + OH^-$	46.35
$[BuSnCl_2(OH)_2]^- \rightarrow BuSnCl(OH)_2 + Cl^-$	6.23
$[BuSnCl_2(OH)_2]^- + H^+ \rightarrow BuSnCl(OH)_2 + HCl$	10.36
$[BuSnCl_2(OH)_2]^- + H^+ \rightarrow BuSnCl_2(OH)(H_2O)$	-6.69
$[BuSnCl_2(OH)_2]^- + OH^- \rightarrow [BuSnCl_2(OH)_3]^{2-}$	-18.48
$[BuSnCl_2(OH)_2]^- + H_2O \rightarrow [BuSnCl_2(OH)_2(H_2O)]^-$	12.95
$2[BuSnCl_2(OH)_2]^- \rightarrow [(BuSn)_2Cl_4(OH)_4]^{2-}$	12.01

Table S3. Possible elementary reactions and corresponding Δ*G* (kcal/mol) in Step 5.1.

Step 5.1	
Reaction	Δ*G*
$BuSnCl_3(H_2O)_2 \rightarrow BuSnCl_3(H_2O) + H_2O$	-9.23
$BuSnCl_3(H_2O)_2 \rightarrow [BuSnCl_2(H_2O)_2]^+ + Cl^-$	14.30
$BuSnCl_3(H_2O)_2 + H^+ \rightarrow [BuSnCl_2(H_2O)_2]^+ + HCl$	18.43

Table S4. Possible elementary reactions and corresponding Δ*G* (kcal/mol) in Step 5.2.

Step 5.2

Reaction	ΔG
$BuSnCl_2(OH)(H_2O) \rightarrow BuSnCl_2(OH) + H_2O$	-2.89
$BuSnCl_2(OH)(H_2O) \rightarrow [BuSnCl_2(H_2O)]^+ + OH^-$	71.25
$BuSnCl_2(OH)(H_2O) \rightarrow [BuSnCl(OH)(H_2O)]^+ + Cl^-$	27.65
$BuSnCl_2(OH)(H_2O) + H^+ \rightarrow [BuSnCl_2(H_2O)_2]^+$	9.39
$BuSnCl_2(OH)(H_2O) + H^+ \rightarrow [BuSnCl(OH)(H_2O)]^+ + HCl$	31.78
$BuSnCl_2(OH)(H_2O) + OH^- \rightarrow [BuSnCl_2(OH)_2(H_2O)]^-$	-36.28
$BuSnCl_2(OH)(H_2O) + H_2O \rightarrow BuSnCl_2(OH)(H_2O)_2$	3.68
$2BuSnCl_2(OH)(H_2O) \rightarrow (BuSn)_2Cl_4(OH)_2(H_2O)_2$	-11.86

Table S5. Possible elementary reactions and corresponding Δ*G* (kcal/mol) in Step 5.3.

Step 5.3	
Reaction	ΔG
$[BuSnCl_2(OH)_3]^{2-} \rightarrow [BuSnCl_2(OH)_2]^- + OH^-$	18.48
$[BuSnCl_2(OH)_3]^{2-} \rightarrow [BuSnCl(OH)_3]^- + Cl^-$	-15.01
$[BuSnCl_2(OH)_3]^{2-} + H^+ \rightarrow [BuSnCl_2(OH)_2(H_2O)]^-$	-24.49
$[BuSnCl_2(OH)_3]^{2-} + H^+ \rightarrow [BuSnCl(OH)_3]^- + HCl$	-10.88

Table S6. Possible elementary reactions and corresponding Δ*G* (kcal/mol) in Step 6.1.

Step 6.1	
Reaction	ΔG
$BuSnCl_3(H_2O) + OH^- \rightarrow [BuSnCl_3(OH)(H_2O)]^-$	-44.55
$BuSnCl_3(H_2O) + H_2O \rightarrow BuSnCl_3(H_2O)_2$	9.23
$BuSnCl_3(H_2O) + H^+ \rightarrow [BuSnCl_2(H_2O)]^+ + HCl$	33.59
$BuSnCl_3(H_2O) \rightarrow [BuSnCl_2(H_2O)]^+ + Cl^-$	29.46
$BuSnCl_3(H_2O) \rightarrow BuSnCl_3 + H_2O$	-4.09

Table S7. Possible elementary reactions and corresponding Δ*G* (kcal/mol) in Step 6.2.

Step 6.2

Reaction	ΔG
$BuSnCl_2(OH) + OH^- \rightarrow [BuSnCl_2(OH)_2]^-$	-46.35
$BuSnCl_2(OH) + H_2O \rightarrow BuSnCl_2(OH)(H_2O)$	2.89
$BuSnCl_2(OH) + H^+ \rightarrow [BuSnCl_2(H_2O)]^+$	18.21
$2BuSnCl_2(OH) \rightarrow (BuSn)_2Cl_4(OH)_2$	-8.64
$(BuSn)_2Cl_4(OH)_2 + 2OH^- \rightarrow [(BuSn)_2Cl_4(OH)_4]^{2-}$	-72.04
$[(BuSn)_2Cl_4(OH)_4]^{2-} + 2H^+ \rightarrow (BuSn)_2Cl_4(OH)_2(H_2O)_2$	-37.25

Table S8. Possible elementary reactions and corresponding Δ*G* (kcal/mol) in Step 6.3.

Step 6.3	
Reaction	Δ*G*
$[BuSnCl_2(OH)_2(H_2O)]^- \rightarrow [BuSnCl_2(OH)_2]^- + H_2O$	-12.95
$[BuSnCl_2(OH)_2(H_2O)]^- \rightarrow BuSnCl_2(OH)(H_2O) + OH^-$	36.28
$[BuSnCl_2(OH)_2(H_2O)]^- \rightarrow BuSnCl(OH)_2(H_2O) + Cl^-$	-5.98
$[BuSnCl_2(OH)_2(H_2O)]^- + H^+ \rightarrow BuSnCl(OH)_2(H_2O) + HCl$	-1.85
$[BuSnCl_2(OH)_2(H_2O)]^- + H^+ \rightarrow BuSnCl_2(OH)(H_2O)_2$	-15.97

Table S9. Possible elementary reactions and corresponding Δ*G* (kcal/mol) in Step 6.4.

Step 6.4	
Reaction	Δ*G*
$[BuSnCl(OH)_3]^- \rightarrow BuSnCl(OH)_2 + OH^-$	39.11
$[BuSnCl(OH)_3]^- \rightarrow BuSn(OH)_3 + Cl^-$	-0.67
$[BuSnCl(OH)_3]^- + H_2O \rightarrow [BuSnCl(OH)_3(H_2O)]^-$	12.18
$[BuSnCl(OH)_3]^- + H^+ \rightarrow BuSn(OH)_3 + HCl$	3.45
$[BuSnCl(OH)_3]^- + H^+ \rightarrow BuSnCl(OH)_2(H_2O)$	-15.46
$2[BuSnCl(OH)_3]^- \rightarrow [(BuSn)_2Cl_2(OH)_6]^{2-}$	0.65

Table S10. Possible elementary reactions and corresponding Δ*G* (kcal/mol) in Step 7.1.

Step 7.1

Reaction	ΔG
$BuSnCl_2(OH)(H_2O)_2 \rightarrow BuSnCl_2(OH)(H_2O) + H_2O$	-3.68
$BuSnCl_2(OH)(H_2O)_2 \rightarrow [BuSnCl_2(H_2O)_2]^+ + OH^-$	61.64
$BuSnCl_2(OH)(H_2O)_2 \rightarrow [BuSnCl(OH)(H_2O)_2]^+ + Cl^-$	17.17
$BuSnCl_2(OH)(H_2O)_2 + H^+ \rightarrow [BuSnCl(OH)(H_2O)_2]^+ + HCl$	21.30
$BuSnCl_2(OH)(H_2O)_2 + H^+ \rightarrow [BuSnCl_2(H_2O)_3]^+$	9.30

Table S11. Possible elementary reactions and corresponding ΔG (kcal/mol) in Step 7.2.

Step 7.2	
Reaction	ΔG
$BuSnCl(OH)_2(H_2O) \rightarrow [BuSnCl(OH)(H_2O)]^+ + OH^-$	69.91
$BuSnCl(OH)_2(H_2O) \rightarrow BuSnCl(OH)_2 + H_2O$	-1.35
$BuSnCl(OH)_2(H_2O) \rightarrow [BuSn(OH)_2(H_2O)]^+ + Cl^-$	26.17
$BuSnCl(OH)_2(H_2O) + OH^- \rightarrow [BuSnCl(OH)_3(H_2O)]^-$	-28.28
$BuSnCl(OH)_2(H_2O) + H_2O \rightarrow BuSnCl(OH)_2(H_2O)_2$	8.67
$BuSnCl(OH)_2(H_2O) + H^+ \rightarrow [BuSn(OH)_2(H_2O)]^+ + HCl$	30.30
$BuSnCl(OH)_2(H_2O) + H^+ \rightarrow [BuSnCl(OH)(H_2O)_2]^+$	7.18
$2BuSnCl(OH)_2(H_2O) \rightarrow (BuSn)_2Cl_2(OH)_4(H_2O)_2$	-9.63

Table S12. Possible elementary reactions and corresponding ΔG (kcal/mol) in Step 7.3.

Step 7.3	
Reaction	ΔG
$BuSn(OH)_3 + OH^- \rightarrow [BuSn(OH)_4]^-$	-28.73
$BuSn(OH)_3 + H_2O \rightarrow BuSn(OH)_3(H_2O)$	3.16
$BuSn(OH)_3 + H^+ \rightarrow [BuSn(OH)_2(H_2O)]^+$	11.39
$2BuSn(OH)_3 \rightarrow (BuSn)_2(OH)_6$	-5.73
$(BuSn)_2(OH)_6 + 2OH^- \rightarrow [(BuSn)_2(OH)_8]^{2-}$	-51.45
$[(BuSn)_2(OH)_8]^{2-} + 2H^+ \rightarrow (BuSn)_2(OH)_6(H_2O)_2$	-56.86

Table S13. Possible elementary reactions and corresponding ΔG (kcal/mol) in Step 8.1.

Step 8.1	
Reaction	ΔG
$BuSnCl(OH)_2 + OH^- \rightarrow [BuSnCl(OH)_3]^-$	-39.11
$BuSnCl(OH)_2 + H_2O \rightarrow BuSnCl(OH)_2(H_2O)$	1.35
$BuSnCl(OH)_2 + H^+ \rightarrow [BuSnCl(OH)(H_2O)]^+$	15.34
$2BuSnCl(OH)_2 \rightarrow (BuSn)_2Cl_2(OH)_4$	-10.09
$(BuSn)_2Cl_2(OH)_4 + 2OH^- \rightarrow [(BuSn)_2Cl_2(OH)_6]^{2-}$	-68.56
$[(BuSn)_2Cl_2(OH)_6]^{2-} + 2H^+ \rightarrow (BuSn)_2Cl_2(OH)_4(H_2O)_2$	-40.12

Table S14. Possible elementary reactions and corresponding Δ*G* (kcal/mol) in Step 8.2.

Step 8.2	
Reaction	ΔG
$[BuSnCl(OH)_3(H_2O)]^- \rightarrow [BuSnCl(OH)_3]^- + H_2O$	-12.18
$[BuSnCl(OH)_3(H_2O)]^- \rightarrow BuSnCl(OH)_2(H_2O) + OH^-$	28.28
$[BuSnCl(OH)_3(H_2O)]^- \rightarrow BuSn(OH)_3(H_2O) + Cl^-$	-9.70
$[BuSnCl(OH)_3(H_2O)]^- + H^+ \rightarrow BuSn(OH)_3(H_2O) + HCl$	-5.57
$[BuSnCl(OH)_3(H_2O)]^- + H^+ \rightarrow BuSnCl(OH)_2(H_2O)_2$	-18.98

Table S15. Possible elementary reactions and corresponding Δ*G* (kcal/mol) in Step 8.3.

Step 8.3	
Reaction	ΔG
$[BuSn(OH)_4]^- \rightarrow BuSn(OH)_3 + OH^-$	28.73
$[BuSn(OH)_4]^- + OH^- \rightarrow [BuSn(OH)_5]^{2-}$	-9.27
$[BuSn(OH)_4]^- + H^+ \rightarrow BuSn(OH)_3(H_2O)$	-24.04
$2[BuSn(OH)_4]^- \rightarrow [(BuSn)_2(OH)_8]^{2-}$	0.29

Table S16. Possible elementary reactions and corresponding Δ*G* (kcal/mol) in Step 9.1.

Step 9.1	
Reaction	ΔG

$BuSn(OH)_3(H_2O) \rightarrow BuSn(OH)_3 + H_2O$	-3.16
$BuSn(OH)_3(H_2O) \rightarrow [BuSn(OH)_2(H_2O)]^+ + OH^-$	64.15
$BuSn(OH)_3(H_2O) + H_2O \rightarrow BuSn(OH)_3(H_2O)_2$	16.47
$BuSn(OH)_3(H_2O) + H^+ \rightarrow [BuSn(OH)_2(H_2O)_2]^+$	2.54
$2BuSn(OH)_3(H_2O) \rightarrow (BuSn)_2(OH)_6(H_2O)_2$	-8.50

Table S17. Possible elementary reactions and corresponding Δ*G* (kcal/mol) in Step 9.2.

Step 9.2	
Reaction	Δ*G*
$BuSnCl(OH)_2(H_2O)_2 \rightarrow BuSnCl(OH)_2(H_2O) + H_2O$	-8.67
$BuSnCl(OH)_2(H_2O)_2 \rightarrow [BuSnCl(OH)(H_2O)_2]^+ + OH^-$	54.44
$BuSnCl(OH)_2(H_2O)_2 \rightarrow [BuSn(OH)_2(H_2O)_2]^+ + Cl^-$	11.82
$BuSnCl(OH)_2(H_2O)_2 + H^+ \rightarrow [BuSn(OH)_2(H_2O)_2]^+ + HCl$	15.95
$BuSnCl(OH)_2(H_2O)_2 + H^+ \rightarrow [BuSnCl(OH)(H_2O)_3]^+$	2.54

Table S18. Possible elementary reaction and corresponding Δ*G* (kcal/mol) in Step 9.3.

Step 9.3	
Reaction	Δ*G*
$[BuSn(OH)_5]^{2-} \rightarrow [BuSn(OH)_4]^- + OH^-$	9.27

Table S19. The Mulliken atomic charge population and Mayer bond order analysis results of $BuSnCl_3$.

Atom	Mulliken Atomic Charge
Sn	1.16
Cl_1	-0.38
Cl_2	-0.39
Cl_3	-0.39
**Bond**	**Mayer Bond Order**
$Sn-Cl_1$	0.90

Sn-Cl_2	0.89
Sn-Cl_3	0.89

Table S20. The Mulliken atomic charge population and Mayer bond order analysis results of $[BuSnCl_3(OH)]^-$.

Atom	Mulliken Atomic Charge
Sn	1.17
Cl_1	-0.62
Cl_2	-0.46
Cl_3	-0.58
O	-0.73
**Bond**	**Mayer Bond Order**
Sn-Cl_1	0.58
Sn-Cl_2	0.81
Sn-Cl_3	0.67
Sn-O	0.84

Table S21. The Mulliken atomic charge population and Mayer bond order analysis results of $[BuSnCl_3(OH)_2]^{2-}$.

Atom	Mulliken Atomic Charge
Sn	1.25
Cl_1	-0.68
Cl_2	-0.77
Cl_3	-0.68
O_1	-0.81
O_2	-0.75
Bond	Mayer Bond Order
Sn-Cl_1	0.53
Sn-Cl_2	0.32

$Sn-Cl_3$	0.52
$Sn-O_1$	0.76
$Sn-O_2$	0.82

Table S22. The Mulliken atomic charge population and Mayer bond order analysis results of $[BuSnCl_2(OH)_2]^-$.

Atom	Mulliken Atomic Charge
Sn	1.26
Cl_1	-0.66
Cl_2	-0.59
O_1	-0.75
O_2	-0.76
**Bond**	**Mayer Bond Order**
$Sn-Cl_1$	0.51
$Sn-Cl_2$	0.67
$Sn-O_1$	0.83
$Sn-O_2$	0.81

Table S23. The Mulliken atomic charge population and Mayer bond order analysis results of $[BuSnCl_2(OH)_3]^{2-}$.

Atom	Mulliken Atomic Charge
Sn	1.36
Cl_1	-0.69
Cl_2	-0.74
O_1	-0.84
O_2	-0.86
O_3	-0.83
**Bond**	**Mayer Bond Order**
$Sn-Cl_1$	0.52

Sn-Cl_2	0.42
Sn-O_1	0.69
Sn-O_2	0.61
Sn-O_3	0.75

Table S24. The Mulliken atomic charge population and Mayer bond order analysis results of $[BuSnCl_2(OH)_2(H_2O)]^-$.

Atom	Mulliken Atomic Charge
Sn	1.30
Cl_1	-0.64
Cl_2	-0.59
O_1	-0.62
O_2	-0.78
O_3	-0.79
**Bond**	**Mayer Bond Order**
Sn-Cl_1	0.53
Sn-Cl_2	0.59
Sn-O_1	0.21
Sn-O_2	0.79
Sn-O_3	0.79

Table S25. The Mulliken atomic charge population and Mayer bond order analysis results of $BuSnCl_2(OH)(H_2O)_2$.

Atom	Mulliken Atomic Charge
Sn	1.26
Cl_1	-0.48
Cl_2	-0.50
O_1	-0.64
O_2	-0.66

O_3	-0.74
Bond	Mayer Bond Order
$Sn\text{-}Cl_1$	0.79
$Sn\text{-}Cl_2$	0.77
$Sn\text{-}O_1$	0.20
$Sn\text{-}O_2$	0.11
$Sn\text{-}O_3$	0.84

Table S26. The Mulliken atomic charge population and Mayer bond order analysis results of $BuSnCl_2(OH)(H_2O)$.

Atom	Mulliken Atomic Charge
Sn	1.27
Cl_1	-0.52
Cl_2	-0.52
O_1	-0.59
O_2	-0.70
Bond	Mayer Bond Order
$Sn\text{-}Cl_1$	0.70
$Sn\text{-}Cl_2$	0.74
$Sn\text{-}O_1$	0.31
$Sn\text{-}O_2$	0.89
